# Supplementary material for: Content-rich biological network constructed by mining PubMed abstracts
Source: BMC Bioinformatics. 2004 Oct 8;5:147. doi: 10.1186/1471-2105-5-147 (PMC528731; doi:10.1186/1471-2105-5-147)
Supplement: Additional File 2 — The original results of the above study (non-essential files are deleted to keep the file size under the limit set by BMC bioinformatics). [file 1471-2105-5-147-S2.bz2 › chilibotAdditionalFile2/dip50/443ID9169459E10419/html/CCL20_CCR6.html]

 


 **CCL20** and **CCR6** 
  
Found 51 abstracts in PubMed, retrieved 50.  
 

 What does Google say? 
 PDF only 
| .edu only 

---

**Interactive relationship** (e.g. stimulation, inhibition, etc)

**Stimulatory relationship**- These findings indicate that increased production of MIP 3 alpha  [ **CCL20** ]  may contribute to the selective recruitment of  **CCR6**  expressing cells in RA.  Ref: 11472439 Clin Exp Immunol, 2001
- In contrast to the starting JK lines, the resulting cell line JK fucosyltransferase VII  **CCR6**  migrated 6 fold better to  **CCL20**  in chemotaxis assays, arrested in response to  **CCL20**  that was immobilized to plastic, and demonstrated a 2.5 fold increase in adhesion to activated HDMEC p = 0.001.  Ref: 12193700 J Immunol, 2002
- Of particular interest is the fact that the enhanced activity on B cells was not associated with an increase in  **CCR6**  expression as assessed by levels of receptor mRNA, surface staining, or MIP 3alpha  **CCL20**  binding sites, or by a change in the affinity of the receptor for ligand.  Ref: 11994436 J Immunol, 2002
- Collectively, our results suggest that epidermal keratinocytes produce LARC  [ **CCL20** ]   **CCL20**  upon stimulation with proinflammatory cytokines such as IL 1alpha and TNF alpha, and attract  **CCR6**  expressing immature dendritic cells and memory effector T cells into the dermis of inflamed skin such as atopic dermatitis.  Ref: 11133838 Int Immunol, 2001
- Treatment of immature DCs with TGF beta 1 resulted in increased expressions of CCR 1, CCR 3, CCR 5, CCR 6  [ **CCR6** ] , and CXC chemokine receptor 4 CXCR 4, which were concomitant with enhanced chemotactic migratory responses to their ligands, RANTES for CCR 1, CCR 3, and CCR 5, macrophage inflammatory protein 3 alpha  [ **CCL20** ]  MIP 3 alpha  [ **CCL20** ]  for CCR 6  [ **CCR6** ] , or stromal cell derived growth factor 1 alpha for CXCR 4.  Ref: 10679062 J Immunol, 2000
**Neutral relationship**- We and others have shown that STRL22  [ **CCR6** ]  is a receptor for the CC chemokine MIP 3 alpha  [ **CCL20** ] , and STRL22  [ **CCR6** ]  has been renamed  **CCR6** .  Ref: 9686174 Braz J Med Biol Res, 1998
- Moreover, calcium signals on T cells in response to MIP 3a  [ **CCL20** ]  were confined to  **CCR6**  expressing cells, consistent with  **CCR6**  being the only MIP 3alpha receptor on peripheral blood T cells.  Ref: 9886385 J Immunol, 1999
- MIP 3alpha  **CCL20**  is the unique chemokine ligand for  **CCR6** , a receptor with a restricted distribution.  Ref: 12760962 Am J Respir Cell Mol Biol< MedlineTA>Am J Respir Cell Mol Biol, 1999
- **CCR6**  is the receptor for the chemokine MIP 3 alpha  [ **CCL20** ]   **CCL20** .  Ref: 12081481 Biochemistry, 2002
- These immature DCs expressed one class of high affinity binding sites for LARC  [ **CCL20** ] , and expressed both  **CCR6**  mRNA and protein.  Ref: 10438902 J Immunol, 1999
- Immature DC respond to many CC and CXC chemokines MIP 1alpha, MIP 1beta, MIP 5, MCP 3, MCP 4, RANTES, TECK, and SDF 1 and in particular to MIP 3alpha LARC  [ **CCL20** ] , which acts through  **CCR6** , a receptor mainly expressed in DC and lymphocytes.  Ref: 10449163 J Leukoc Biol, 1999
- Macrophage inflammatory protein 3alpha  **CCL20**  is a recently identified chemokine that binds to  **CCR6**  and acts as a chemoattractant for memory differentiated T cells, B cells, and immature dendritic cells.  Ref: 12528183 Glia, 2003
- A close association was observed between  **CCL20**  producing cells and CD1a cells, suggesting the contribution of  **CCL20**  to  **CCR6**  cell homing.  Ref: 11994492 J Immunol, 2002
- Only on follicular B cells the cognate receptor for  **CCL20** , CC chemokine receptor 6  [ **CCR6** ]   **CCR6** , was expressed strongly and a functional transmigration assay with  **CCR6**  negative B cells demonstrated conclusively the sole signaling of  **CCL20**  through  **CCR6** .  Ref: 12816871 Blood< MedlineTA>Blood, 2002
- **CCL20**  is a CC type chemokine that binds to and activates CC chemokine receptor 6  [ **CCR6** ]   **CCR6** .  Ref: 12149255 J Biol Chem, 2002
- Similar structural features are seen in human beta defensin 2, a small nonchemokine polypeptide reported to selectively bind and activate  **CCR6** , which stresses their importance for the specific binding of both  **CCL20**  and beta defensin 2 to  **CCR6** .  Ref: 11373289 J Biol Chem, 2001
- **CCR6**  is the only known receptor for the chemokine macrophage inflammatory protein MIP 3alpha CC chemokine ligand CCL 20  [ **CCL20** ] .  Ref: 11994436 J Immunol, 2002
- CC chemokine receptor 6  [ **CCR6** ]  is expressed on diverse memory subsets of T cells and determines responsiveness to macrophage inflammatory protein 3 alpha  [ **CCL20** ] .  Ref: 9886385 J Immunol, 1999
- Preincubation with the  **CCR6**  ligand  **CCL20**  induced liver sorted tumour cells to preferentially colonize the liver, demonstrating an association between liver metastasis and  **CCR6**  expression in the mouse.  Ref: 12791091 Scand J Immunol, 2003
- Cytokine activated PMNLs expressed functional  **CCR6** , as detected by the binding of sodium iodide I 125 labeled liver and activation regulated chemokine  [ **CCL20** ]  LARC  [ **CCL20** ]  and dose dependent migration toward LARC  [ **CCL20** ] .  Ref: 11090084 Blood, 2000
- Macrophage inflammatory protein 3alpha MIP 3alpha  **CCL20**  and MIP 3beta CCL19 are members of the CC chemokine subfamily which exert their effects through specific receptors,  **CCR6**  and CCR7, respectively.  Ref: 11449350 Eur J Immunol, 2001
- Liver and activation regulated chemokine  [ **CCL20** ]  LARC  [ **CCL20** ] , also designated macrophage inflammatory protein 3alpha MIP 3alpha, Exodus, or  **CCL20** , is a C C chemokine that attracts immature dendritic cells and memory T lymphocytes, both expressing  **CCR6** .  Ref: 11035086 J Immunol, 2000
- NMR solution structure of murine  **CCL20**  MIP 3alpha, a chemokine that specifically chemoattracts immature dendritic cells and lymphocytes through its highly specific interaction with the beta chemokine receptor  **CCR6** .  Ref: 11373289 J Biol Chem, 2001
- **CCL20**  MIP 3alpha is a beta chemokine expressed in the thymus, skin, and intestinal epithelial cells that exclusively binds and activates the  **CCR6**  receptor in both mice and humans.  Ref: 11373289 J Biol Chem, 2001
- Liver and activation regulated chemokine  [ **CCL20** ]  LARC  [ **CCL20** ]  macrophage inflammatory protein MIP 3alpha  **CCL20**  is a CC chemokine which is constitutively expressed by follicle associated epithelial cells in the mucosa, and attracts cells expressing  **CCR6**  such as immature dendritic cells and alpha 4 beta 7 high intestine seeking memory T cells.  Ref: 11133838 Int Immunol, 2001
- The generality of the latter finding was demonstrated by the fact that  **CCL20**   **CCR6**  interactions increase the basal proliferation rate of CD62L lo CD4 T cells irrespective of their thymic in non OM transgenic mice or extrathymic in LckOM mice origin.  Ref: 12702501 Blood< MedlineTA>Blood, 2001
- Chemokine ligand 20  [ **CCL20** ]   **CCL20**  and human beta defensins HBDs share structural and functional properties, including anti parallel beta pleated sheet core structures, charge distribution, and signaling to adaptive immune cells via the highly selective  **CCR6**  receptor.  Ref: 12748059 Am J Respir Cell Mol Biol< MedlineTA>Am J Respir Cell Mol Biol, 2001
- Liver and activation regulated chemokine  [ **CCL20** ]  LARC  [ **CCL20** ]   **CCL20**  is expressed by surface lining epithelial and epidermal cells, and is likely to link innate and acquired immunity by attracting immature dendritic cells, effector memory T cells and B cells via  **CCR6** .  Ref: 11581170 Int Immunol, 2001

**Non-interactive relationship** (e.g. studied together, co-existance, homology, etc.)

- In contrast to other chemokines, including MCP 1 and IL 8, the natural processing did not affect the calcium mobilizing capacity of LARC  [ **CCL20** ]  MIP 3alpha through its receptor  **CCR6** .  Ref: 11035086 J Immunol, 2000
- Strikingly, desensitization of  **CCR6**  with LARC  [ **CCL20** ] , but not thymus and activation regulated chemokine CCL17 or secondary lymphoid tissue chemokine CCL21, caused a 50 75% decrease p <.  Ref: 11120783 J Immunol, 2000
- We then focused on the expression of  **CCL20**   **CCR6**  and CCL19 CCR7, CCL21 CCR7 chemokine receptor complex, which control immature and mature DC migration respectively.  Ref: 11994492 J Immunol, 2002
- In mice, intradermal injection of IL 1alpha and TNF alpha rapidly induced a local accumulation of transcripts for LARC  [ **CCL20** ]   **CCL20**  and its receptor  **CCR6**  with a lag of several hours in the latter.  Ref: 11133838 Int Immunol, 2001
- Recombinant liver and activation regulated chemokine  [ **CCL20** ]  LARC  [ **CCL20** ]   **CCL20**  a  **CCR6**  ligand induced firm arrest of cutaneous lymphocyte Ag mTC in a flow chamber system using purified substrates.  Ref: 11120783 J Immunol, 2000
- MIP 3alpha  **CCL20**  induces selective migration of DCs because  **CCR6**  is expressed on some immature DCs but not on CD14 DC precursors or mature DCs.  Ref: 12760962 Am J Respir Cell Mol Biol< MedlineTA>Am J Respir Cell Mol Biol, 2000
- Ligation by TNF alpha resulted in down modulation of cell surface expressions of CCR 1, CCR 3, CCR 5, CCR 6  [ **CCR6** ] , and CXCR 4, and the chemotaxis for RANTES, MIP 3 alpha  [ **CCL20** ] , and stromal cell derived growth factor 1 alpha.  Ref: 10679062 J Immunol, 2000
- Furthermore, we have identified CCR4 as the specific receptor for TARC, GPR CY4  [ **CCR6** ]  DRY6  [ **CCR6** ]  CKR L3  [ **CCR6** ]  STRL22  [ **CCR6** ]  as that for LARC  [ **CCL20** ]   **CCR6** , and EBI1 BLR2 as that for ELC CCR7.  Ref: 9365118 J Leukoc Biol, 1997
- Up regulation of macrophage inflammatory protein 3 alpha  [ **CCL20** ]   **CCL20**  and CC chemokine receptor 6  [ **CCR6** ]  in psoriasis.  Ref: 10843722 J Immunol, 2000
- These data suggest that restricted patterns of  **CCR6**  expression and MIP 3alpha  **CCL20**  responsiveness are integral parts of the process of B lineage maturation and antigen driven B cell differentiation.  Ref: 11001880 Blood, 2000
- 3 in concordance with the sequential expression of CCR2 and  **CCR6** , the HPC derived CD14 precursors initially acquire the ability to migrate in response to MCP 4 CCL13 and subsequently in response to MIP 3alpha  **CCL20** .  Ref: 11782014 Eur J Immunol, 2002
- Human B cells become highly responsive to macrophage inflammatory protein 3 alpha  [ **CCL20** ]  CC chemokine ligand 20  [ **CCL20** ]  after cellular activation without changes in  **CCR6**  expression or ligand binding.  Ref: 11994436 J Immunol, 2002
- In addition, IL 10 induced the expression of  **CCR6**  and responsiveness to  **CCL20**  in differentiated monocytes that preserve their ability to differentiate into mature DC.  Ref: 11698430 J Immunol, 2001
- Furthermore, regardless of the tissue site, LCH lesions markedly overexpressed  **CCL20**  MIP 3alpha, the ligand for  **CCR6** .  Ref: 12743170 J Exp Med, 2003
- In this study, we investigated the effects of cytokines on the expression of the  **CCL20**  receptor,  **CCR6** , during differentiation of LC.  Ref: 11698430 J Immunol, 2001
- The chemokine receptor  **CCR6**  is unusual in that it mediates leukocyte chemotaxis in response to a single chemokine,  **CCL20**  macrophage inhibitory factor 3alpha, as well as in response to a family of antimicrobial peptides termed beta defensins.  Ref: 12514792 Hum Pathol, 2002
- Dual label confocal microscopy revealed that LFA 1 CD18 and CD11a, but not CXCR4, colocalized with clustered  **CCR6**  in the presence of immobilized  **CCL20** .  Ref: 12193700 J Immunol, 2002
- We have previously reported that  **CCL20**  and  **CCR6**  mRNAs are expressed in the CNS of SJL mice with experimental autoimmune encephalomyelitis EAE.  Ref: 12528183 Glia, 2003
- We found that both IL 4 and IFN gamma blocked the expression of  **CCR6**  and  **CCL20**  responsiveness at different stages of LC development.  Ref: 11698430 J Immunol, 2001
- For examples, Langerhans cells migrate selectively to MIP 3 alpha  [ **CCL20** ]  via  **CCR6** , blood CD11c DC to MCP chemokines via CCR2, monocytes derived DC respond to MIP 1 alpha beta via CCR1 and CCR5, while blood CD11c DC precursors do not respond to any of these chemokines.  Ref: 11155441 Springer Semin Immunopathol, 2000
- Desensitization of  **CCR6**  on naive B cells with  **CCL20**  resulted in receptor downregulation and reduced B cell adhesion.  Ref: 12816871 Blood< MedlineTA>Blood, 2000
- Selective recruitment of  **CCR6**  expressing cells by increased production of MIP 3 alpha  [ **CCL20** ]  in rheumatoid arthritis.  Ref: 11472439 Clin Exp Immunol, 2001
- Activated cells are thought to down regulate  **CCR6** , whose ligand macrophage inflammatory protein 3 alpha  [ **CCL20** ]  MIP 3 alpha  [ **CCL20** ]   **CCL20**  is expressed in skin, and up regulate CCR7, whose ligands are in lymphoid tissues.  Ref: 12642342 Blood, 2003
- During the extrafollicular response, plasmablast expression of CXCR5 and responsiveness to B lymphocyte chemoattractant CXCR5 as well as to secondary lymphoid tissue chemokine CCR7 and stromal cell derived factor SDF 1 CXCR4 are lost while a weak response towards the  **CCR6**  chemokine LARC  [ **CCL20** ]  is maintained.  Ref: 11180126 Eur J Immunol, 2001
- Therefore, LARC  [ **CCL20** ]   **CCR6**  interaction presumably also contributes to the regulation of trafficking of monocyte derived DCs, and utilization of TGF beta can potentially provide a ready source of  **CCR6**  monocyte derived DCs for therapeutic purposes.  Ref: 10438902 J Immunol, 1999
- The binding of iodinated LARC  [ **CCL20** ] , the chemokine ligand for  **CCR6** , to  **CCR6**  transfected cells was competitively displaced by beta defensin.  Ref: 10521347 Science, 1999
- Detection and localization of Mip 3alpha LARC  [ **CCL20** ]  Exodus, a macrophage proinflammatory chemokine, and its  **CCR6**  receptor in human pancreatic cancer.  Ref: 10225458 Int J Cancer, 1999
- Taken together, these findings strongly suggest that  **CCL20**   **CCR6**  may play a role in the recruitment of T cells to lesional psoriatic skin.  Ref: 10843722 J Immunol, 2000
- Because human liver constitutively expresses  **CCL20** , it could attract and select  **CCR6**  cancer cells.  Ref: 12791091 Scand J Immunol, 2003
- In addition, 3 6 cell lines migrated in response to MIP 3 alpha  [ **CCL20** ]  and IP 10, ligands for  **CCR6**  and CXCR3, respectively.  Ref: 12529679 Leukemia, 2003
- Regulation of  **CCR6**  chemokine receptor expression and responsiveness to macrophage inflammatory protein 3alpha  **CCL20**  in human B cells.  Ref: 11001880 Blood, 2000
- In contrast to other chemokines, such as monocyte chemotactic protein 1 CC chemokine receptor 2 CCR2, macrophage inflammatory protein 1beta CCR5, liver and activation regulated chemokine  [ **CCL20** ]  LARC  [ **CCL20** ]   **CCR6** , Epstein Barr virus induced molecule 1 ligand chemokine ELC CCR7, and IP10 CXCR3, SDF 1 stimulates the prolonged activation of protein kinase B and extracellular signal regulated kinase ERK 2.  Ref: 10934220 J Exp Med, 2000
- In this study, we investigated the expression of MIP 3 alpha  [ **CCL20** ]  and its specific receptor  **CCR6**  in the inflamed joints of patients with RA.  Ref: 11472439 Clin Exp Immunol, 2001
- Expression of the specific receptor of MIP 3 alpha  [ **CCL20** ] ,  **CCR6** , was also increased in HTLV I infected T cell lines, suggesting an autocrine and or paracrine mechanism to establish the pathogenesis of HTLV I associated diseases.  Ref: 11809734 Int Immunol, 2002
- SIV infection altered the expression of chemokines CCL19 MIP 3beta, CCL21 6Ckine, and  **CCL20**  MIP 3alpha and of chemokine receptors CCR7 and  **CCR6**  that drive DC trafficking.  Ref: 12406887 Blood, 2003
- The increased expression of CCR5 resulted in enhanced chemotaxis toward the CCR5 ligand macrophage inflammatory protein 1beta CCL4, but up regulation of  **CCR6**  did not result in altered chemotactic responsiveness to macrophage inflammatory protein 3alpha  **CCL20** , suggesting an alternative function for this receptor.  Ref: 11751947 J Immunol, 2002
- We report here that the CC chemokine, macrophage inflammatory protein 3 alpha  [ **CCL20** ] , recently renamed  **CCL20** , and its receptor  **CCR6**  are markedly up regulated in psoriasis.  Ref: 10843722 J Immunol, 2000
- Our results suggest that LARC  [ **CCL20** ]  production by activated endothelial cells and  **CCR6**  expression by mTC may be critical components in the pertussis toxin sensitive arrest of mTC on activated HDMEC.  Ref: 11120783 J Immunol, 2000
- CCL19 MIP 3beta,  **CCL20**  MIP 3alpha,  **CCR6** , and CCR7 expression increased in lymph nodes during the early systemic burst of viral replication acute infection.  Ref: 12406887 Blood, 2003
